# Dietary Habits and Lifestyle Characteristics of Automotive Sector Workers: An Observational Study

**DOI:** 10.7759/cureus.91164

**Published:** 2025-08-28

**Authors:** Cansu Gingir, Sultan Pınar Çetintepe, Esra Gultekin Koc, Volkan Medeni

**Affiliations:** 1 Directorate of Health Services, Kilis Provincial Health Directorate, Kilis, TUR; 2 Department of Occupational Medicine, Gazi University Faculty of Medicine, Ankara, TUR; 3 Department of Public Health, Gazi University Faculty of Medicine, Ankara, TUR

**Keywords:** dietary habits, environmental and occupational health, food and nutrition, hard work, lifestyle habits

## Abstract

Introduction

Hazardous working conditions in the automotive sector emphasize the importance of dietary and lifestyle habits among workers. This study investigates the nutritional preferences and lifestyle habits of automotive sector workers in Turkey.

Methods

The descriptive study was conducted at Ankara Occupational and Environmental Diseases Hospital. The participants were employees from automotive factories undergoing periodic health examinations in 2022. Data on sociodemographic information, body mass index, chronic diseases, medication use, lifestyle characteristics, and dietary habits were assessed using a questionnaire and medical records.

Results

All participants were men. The participants had a mean age of 38.0 years, and 102 (65.8%) of them were pre-obese or obese. Eighteen (11.6%) participants had a chronic disease. Twenty-three (14.8%) people regularly used prescription drugs. Forty-nine (31.6%) participants had a hobby. Smoking prevalence was high, with 96 (61.9%) people. Weekly red meat consumption was frequent, with 152 (98.1%) of them, and 117 (77.0%) people preferred well-cooked meat. Poultry and fish consumption was lower, with 32 (20.6%) and 47 (30.3%) of the participants not consuming them weekly, respectively. Vegetable consumption was typical, with 130 (83.9%) people, but water drinking was inadequate, with only 37 (23.9%) of the participants drinking eight or more glasses of water daily. Tea and coffee were consumed regularly, with 82 (52.9) and 109 (70.3) of the participants, respectively. Eight (5.2%) of the participants were taking vitamin supplements.

Conclusion

The study highlights the high prevalence of overweight, smoking, and unhealthy dietary habits among automotive sector workers. Strategies to promote healthy lifestyles, such as nutrition education, smoking cessation programs, and improving access to drinking water, are needed to enhance employee health and well-being in this sector. Further research is warranted to explore the factors influencing dietary habits and develop targeted interventions.

## Introduction

The manufacturing sector is the branch of industry in which raw materials to be used in production are transformed into intermediate goods by processing with the help of manual labor or machines. It covers various sectors such as automotive, textile, machinery, and chemistry [1]. Globally, the manufacturing sector drives economic development by contributing significantly to employment and technological innovations [2]. According to the World Bank's 2022 data, the manufacturing sector's contribution to Turkey's gross domestic product was 22% [3]. The automotive manufacturing sector is recognized as one of the largest industries in the world, with the production of approximately 70 million vehicles each year. This sector also employs 14 million people worldwide [4]. In Turkey, 56,722 employees are used in the automotive manufacturing sector [5].

The hazardous working conditions of automotive sector employees, which are based on physical strength and require mental alertness and concentration, bring the importance of nutrition to the agenda. Adopting a healthy lifestyle and improving the nutritional status of employees are critical factors in protecting performance and general health [6]. Irregular working hours and shift patterns in this sector may lead to changes in eating habits [7].

Nutritional habits of employees play a fundamental role in their health and overall quality of life and affect their health and productivity levels in the workplace. Unhealthy nutrition may lead to fatigue, decreased cognitive functions, and increased risk of chronic health problems, negatively affecting workplace safety [8]. In the short term, favoring nutritious and balanced meals may reduce stress, prevent accidents, and improve attention levels, memory, learning, and overall productivity. The consumption of unhealthy foods may lead to obesity and chronic diseases in employees, and macro- and micronutrient deficiencies may lead to malnutrition [9]. Maintaining nutritional balance in the long term is directly related to improving the nutritional status of individuals and societies. It plays a vital role in improving the quality of life and reducing the prevalence of noncommunicable diseases [10].

The role of nutrition in preventing diseases and reducing occupational accidents is indisputable. The automotive sector is a hazardous line of work with physically demanding tasks, fast-paced working, and high risk of ergonomics, and the nutritional status of workers directly affects general health and work performance. Examining the dietary habits of automotive sector workers can contribute to identifying areas requiring intervention and developing healthy living programs in the workplace. This study aimed to investigate automotive sector workers' lifestyle habits, such a smoking and alcohol consumption status, and dietary habits, such as meat and poultry consumption and nutritional preferences.

## Materials and methods

This descriptive study was conducted at Ankara Occupational and Environmental Diseases Hospital, a unique national reference center for occupational diseases. The population consisted of employees in the production department of automotive factories who were sent to the hospital for periodic health examinations in 2022. Ethical approval was obtained from the Gazi University Ethics Commission (approval number: 2024-285). Participation was entirely voluntary, and informed consent was collected from each participant according to the principles of the Declaration of Helsinki. All automotive sector employees who came to the hospital were included in the study. The study was conducted with 155 participants.

Sociodemographic information such as age, marital status, graduation status, occupation, total working time, and weekly working hours was questioned. The questionnaire was first applied to 15 participants as a pilot test. It was determined that no changes were needed after the feedback, so the 15 pilot tests were added to the total number. All questionnaires were conducted by the same physician. Body mass index, the presence of chronic diseases, regular medication intake, the use of vitamin supplements and sweeteners, lifestyle characteristics, and dietary habits were examined.

The height and body weight of the patients were measured at the outpatient clinic during the medical examination with standard clinical equipment belonging to the same physician. The body mass index was calculated by dividing the weight by the square of the height. Considering the obesity classification of the World Health Organization, the average value was accepted as 18.50-24.99 kg/m^2^, pre-obesity as 25.00-29.99 kg/m^2^, and obesity as above 30.00 kg/m^2^ [11]. Chronic diseases refer to diseases diagnosed by a doctor. The health records of the participants were analyzed and evaluated. Under the heading of lifestyle, hobbies, smoking, chewing tobacco, and alcohol use were included, with hobbies defined as leisure-time activities outside of work. The data on smoking, chewing tobacco, and alcohol use represent point prevalences and were obtained based on the participants' statements.

Data on the weekly consumption of red meat, poultry meat, fish meat, and vegetables; the cooking style of red meat; and the daily frequency of drinking water, tea, and coffee were collected. To evaluate the dietary habits of the employees, the participants were asked relevant questions by a dietician during the periodic health examination. The questions about the frequency of drinking water, tea, and coffee were prepared by considering the Turkish Nutrition and Health Survey. The participants were told that a standard water glass is 200 mL, a tea glass is 100 mL, and a coffee cup is 250 mL.

Data analysis was performed using IBM's SPSS 25.0 program (IBM Corp., Armonk, NY). Descriptive statistics for categorical variables were calculated as numbers (n) and percentages (%). Continuous variables were presented using mean, standard deviation, and minimum and maximum values.

## Results

One hundred fifty-five employees participated in our research. All of the participants were men. The mean age was 38.0±9.9 years (minimum, 23; maximum, 61), the mean height was 174.7±6.3 cm (minimum, 156; maximum, 192), the mean body weight was 81.6±11.6 kg (minimum, 55; maximum, 110), and the mean body mass index was 26.8±3.7 kg/m^2^ (minimum, 18.3; maximum, 36.7). The mean duration of employment in the sector was 11.9±9.0 years (minimum, two; maximum, 39), and the mean weekly working time was 45.3±4.9 hours (minimum, 36; maximum, 63).

Eighty-six (55.5%) participants are under 40 years of age. One hundred twenty-nine (83.2%) of them are married. Ninety-six (62.0%) of them graduated from high school or higher-level educational institutions. One hundred (64.6%) of them are painters, and 25 (16.1%) of them are clerical staff. Eighty-six (55.5%) participants have worked in the sector for less than 10 years. Sixty-seven (43.3%) of them work 45 hours per week, and 58 (37.3%) participants work more than 45 hours per week (Table 1).

**Table 1:** Personal and Occupational Characteristics of the Participants (Turkey, 2022). *Janitor and security personnel

	Number (n)	Percentage (%)
Age (n=155)		
Less than 30 years old	44	28.4
30-39 years old	42	27.1
40-49 years old	44	28.4
50 years and older	25	16.1
Marital status (n=155)		
Married	129	83.2
Single	23	14.9
Divorced	3	1.9
Graduation (n=155)		
Primary school	32	20.6
Middle school	27	17.4
High school	77	49.7
University	19	12.3
Occupation (n=155)		
Painter	100	64.6
Office staff	25	16.1
Welder	9	5.8
Sander	7	4.5
Driver	5	3.2
Quality control	4	2.6
Operator	3	1.9
Others*	2	1.3
Total working time (n=155)		
5 years and less	52	33.5
6-10 years	34	22.0
11-20 years	42	27.1
More than 20 years	27	17.4
Weekly working hours (n=155)		
40 hours	30	19.4
45 hours	67	43.3
More than 45 hours	58	37.3

**Figure 1:**
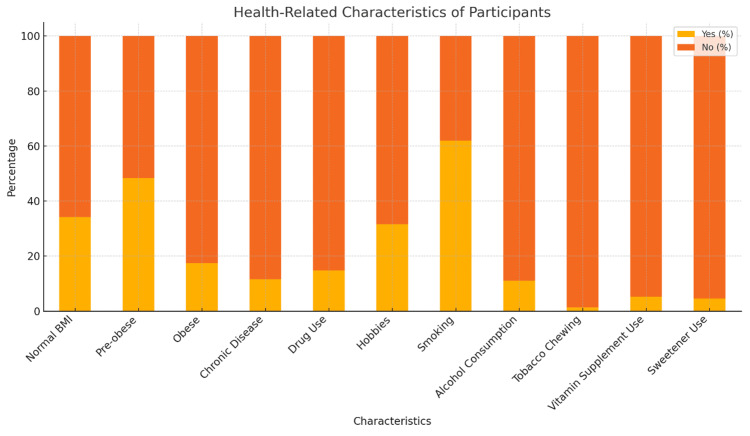
Health-Related Characteristics of the Participants (Turkey, 2022). BMI: body mass index

The body mass index category of 102 (65.8%) participants in our study was pre-obese or obese (Figure 1). Eighteen (11.6%) participants had a chronic disease. Twenty-three (14.8%) of them regularly used prescription drugs. Forty-nine (31.6%) participants had a hobby. Ninety-six (61.9%) of the participants smoked cigarettes, and 17 (11.0%) of them consumed alcohol. Eight (5.2%) participants used vitamin supplements, and seven (4.5%) participants used sweeteners (Table 2).

**Table 2:** Health-Related Characteristics of the Participants (Turkey, 2022).

	Number (n)	Percentage (%)
Body mass index (n=155)		
Normal	53	34.2
Pre-obese	75	48.4
Obese	27	17.4
Chronic disease (n=155)		
Yes	18	11.6
No	137	88.4
Drug use (n=155)		
Yes	23	14.8
No	132	85.2
Hobbies (n=155)		
Yes	49	31.6
No	106	68.4
Smoking (n=155)		
Yes	96	61.9
No	59	38.1
Alcohol consumption (n=155)		
Yes	17	11.0
No	138	89.0
Tobacco chewing (n=155)		
Yes	2	1.3
No	153	98.7
Use of vitamin supplements (n=155)		
Yes	8	5.2
No	147	84.8
Sweetener use (n=155)		
Yes	7	4.5
No	148	85.5

**Figure 2:**
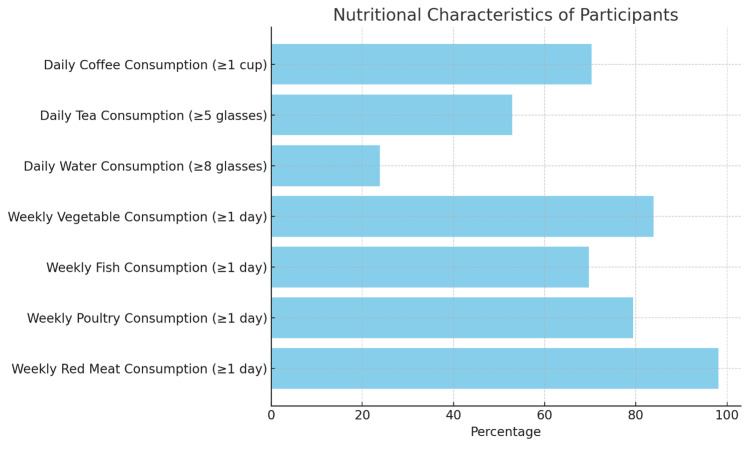
Nutritional Characteristics of the Participants (Turkey, 2022).

In our study, 152 (98.1%) of the participants consume red meat at least one day a week. Of those who consume red meat, 117 (77.0%) people eat well-cooked beef. Thirty-two (20.6%) of the participants do not consume poultry meat, and 47 (30.3%) of them do not consume fish meat during the week (Figure 2). One hundred thirty (83.9%) of the respondents eat vegetable meals at least one day a week. Thirty-seven (23.9%) of the participants consumed eight or more glasses of water daily. Eighty-two (52.9%) of the participants drank more than five cups of tea, and 109 (70.3%) participants drank at least one cup of coffee daily (Table 3).

**Table 3:** Nutritional Characteristics of the Participants (Turkey, 2022).

	Number (n)	Percentage (%)
Weekly red meat consumption (n=155)		
None	3	1.9
Less than one day	29	18.7
1-2 days	53	34.2
3-4 days	37	23.9
5-6 days	33	21.3
Red meat cooking preference (n=152)		
Undercooked	6	4.0
Medium cooked	29	19.0
Well-cooked	117	77.0
Weekly poultry meat consumption (n=155)		
None	32	20.6
Less than one day	38	24.5
1-2 days	63	40.7
3-4 days	19	12.3
5-6 days	3	1.9
Weekly fish meat consumption (n=155)		
None	47	30.3
Less than one day	60	38.7
1-2 days	30	19.4
3-4 days	16	10.3
5-6 days	2	1.3
Weekly vegetable consumption (n=155)		
None	12	7.7
Less than one day	13	8.4
1-2 days	67	43.3
3-4 days	34	22.0
5-6 days	29	18.6
Daily water drinking (n=155)		
Less than one glass	4	2.6
1-2 glasses	36	23.2
3-4 glasses	40	25.8
5-7 glasses	38	24.5
8 or more glasses	37	23.9
Daily tea drinking (n=155)		
Less than one glass	8	5.2
1-2 glasses	19	12.2
3-4 glasses	46	29.7
5-7 glasses	49	31.6
8 or more glasses	33	21.3
Daily coffee drinking (n=155)		
Less than one cup	46	29.7
1 cup	86	55.5
2 cups	18	11.6
3 cups	5	3.2

## Discussion

This study's results reveal the participants' overall profile and provide an essential insight into their health habits, dietary preferences, and lifestyle factors. In this study, approximately half of the participants were pre-obese, and one-sixth were obese. In a study conducted in South Africa in the automotive manufacturing sector and investigating cardiovascular risk factors in the automotive industry, two-thirds of the participants were classified as pre-obese or obese, and the prevalence of obesity was high among shift workers [12]. In a study conducted in Brazil, the overall prevalence of obesity among industrial workers was found to be one in seven, and increasing age, being married, low education level, and smoking history were defined as factors associated with obesity [13]. These findings show that being overweight is high among automotive sector workers. The reason for this may be the preference for unhealthy foods such as fast food due to intense and strenuous working conditions, decreased regular physical activity, or the emergence of emotional eating tendencies due to work stress.

In this study, it was observed that more than three-fifths of automotive sector workers smoked. Turkey is among the countries with a high prevalence of smoking, and 41.3% of men smoke every day [14]. In a study conducted in South Korea, 56.1% of men working in the manufacturing industry smoked daily [15]. In a study conducted in an automobile manufacturing company in Iran, the prevalence of smoking among men was found to be 20.5%. It was stated that occupational and non-occupational stress factors were associated with smoking, and non-occupational stress factors had a more significant effect [16]. Cultural factors may be the main reason for the difference between frequencies. Social norms and group pressure among employees in the sector may also increase smoking habits, and smoking may be considered a part of the work culture. The workload that employees in the automotive industry are exposed to may also affect the prevalence of smoking habits. The combination of all these reasons may have led to the overall high prevalence of smoking in our study.

Approximately one-third of the employees participating in the study consume red meat 1-2 days a week, one-fourth 3-4 days a week, and one-fifth 5-6 days a week. The prevalence of red meat consumption is affected by various factors such as income, religious beliefs, regional conditions, education level, and household size [17]. Turkey's red meat consumption is approximately half the world's average [18]. It was found that only 29.1% of consumers consume enough red meat to meet the criteria for a balanced diet [19]. A study conducted in Turkey found that low-income participants consumed less red meat than high-income participants [20]. Another study found that red meat consumption in the household decreased if the breadwinner was a blue-collar worker [21]. Socioeconomic factors may have a significant effect on red meat consumption. Our study's participants consume more red meat than the general Turkish population. Because the participants work in heavy industry and under harsh conditions, red meat is probably served almost daily in their workplaces.

In this study, three-quarters of heavy industry workers consumed well-cooked meat. Many studies show that high meat consumption, especially overcooked red meat and processed meat, may increase some cancer risks [22]. Heterocyclic amines and polycyclic aromatic hydrocarbons are chemicals formed by cooking meat using high-temperature methods and have been found to create a substructure for cancer development [23]. In light of this information, our results emphasize the necessity of increasing health awareness and encouraging healthy eating habits among our participants.

One-fifth of the respondents do not consume poultry meat, and one-third do not consume fish meat. Most of those who consume poultry meat eat it 1-2 days a week, and most of those who consume fish meat eat it less than once a week. In a study on white meat consumption conducted in Turkey, 18% of the participants who consumed white meat said that they consumed it rarely, 4% every day, 40% once a week, and 38% at least twice a week [24]. According to a study conducted in coastal regions of Turkey, 81% of the participants consumed fish, and 63% ate fish meat once a week [25]. According to another study conducted in a coastal city in Turkey, the frequency of fish consumption was determined as once a week by 33.3%, once a month by 21.5%, and once every two months by 21.9%, and 2.3% of the participants stated that they never consumed fish [26]. All our participants lived in Ankara, the capital city of Turkey. Ankara is located in the inland region, where it is challenging to access seafood. Therefore, people may consume less fish because accessing fresh and diverse fish sources may take more work. Cultural preferences, economic factors, and the awareness of the health benefits of white meat may also be among the reasons for the low consumption of poultry and fish meat.

In this study, more than four-fifths of the participants consumed vegetables more than one day a week. In a study conducted in Spain, the overall prevalence of daily vegetable consumption among workers was 40% [27]. A study conducted in Brazil showed that industrial workers, men, and individuals with less than a high school education had a lower prevalence of vegetable consumption [28]. Turkey ranks eighth worldwide in vegetable consumption [29]. The fact that Turkey generally has a Mediterranean-type diet may favor vegetable consumption among our participants. Turkish cuisine is recognized for its various dishes with plenty of vegetables. It may be a reflection of local culture. In addition, Turkey's climate and soil structure allow for a wide variety of vegetables, which may make them accessible and affordable.

This study determined that three-fifths of the automotive sector employees drink 3-7 cups of tea, and more than half of them drink one cup of coffee daily. According to our findings, more than three-quarters of our participants need to drink more water. Tea consumption is typical in Turkey, and black tea is one of the most consumed beverages [30,31]. A study conducted in China showed that regular tea consumption by industrial workers led to a decrease in oxidative stress with an increase in total antioxidant capacity and thiol group levels [32]. Most participants drink less water because they consume a lot of tea and coffee during the day. However, it is necessary to evaluate the water consumption habits of employees in more detail, considering that adequate water consumption is vital to ensure the proper functioning of bodily functions, especially for those who work in physically active jobs. Only 10% of them stated that they consumed alcohol. It may be due to social desirability bias, as alcohol consumption may not be seen as a good behavior in Turkey.

Although our participants consumed high amounts of tea and coffee, most did not take vitamin supplements. Vitamin supplements are generally recommended for people with high tea and coffee consumption because these drinks may increase water excretion from the body and decrease the absorption of some vitamins. The excessive consumption of caffeine-containing beverages may significantly affect the absorption of B vitamins [33,34]. Vitamin levels should be checked regularly to determine vitamin deficiency in these workers. In case of need, an appropriate vitamin supplement may be recommended, taking into account the dietary habits and lifestyle of the person.

Overall, these findings can be interpreted within the framework of social determinants of health, as working conditions, lifestyle behaviors, and socioeconomic factors can shape health outcomes. In this context, the high prevalence of unhealthy dietary patterns of automotive sector employees not only reflects personal choices but also underlines the need for workplace health promotion strategies.

This study has some limitations. Due to the small number of participants and the study's descriptive, observational design, the results are unsuited for generalization and establishing a causal relationship. Nevertheless, all automotive sector workers admitted to the only occupational disease hospital in Turkey throughout the year were included in our study, but the sample may not represent the broader workforce. Most of the data are based on self-reported information, which may lead to uncertainties about the reliability of the responses. However, the fact that body mass index was based on direct measurements of the participants is also a strength of our study. Also, participants being only men is an important limitation, as female workers could have different dietary and lifestyle choices. The lack of questionnaire validation and inferential statistics limits the exploration of the associations between variables. Future analytical research controlling for confounders such as gender, income level, shift patterns, and job stress is needed to better understand the relationship between dietary habits, lifestyle factors, and health outcomes in automotive sector employees.

## Conclusions

The findings reveal a high prevalence of pre-obesity and obesity among participants, alongside unhealthy habits such as consuming well-cooked meat, inadequate water intake, high smoking rates, and low vitamin consumption. Improving the nutritional awareness and lifestyle habits of employees in the automotive sector is crucial for enhancing health services and quality of life. Workplace initiatives could include exercise programs, healthy nutrition support, and serving medium-cooked red meat. Awareness campaigns on the health impact of dietary habits, smoking cessation programs, and increased access to drinking water may further support these efforts. Additionally, comprehensive research is needed to explore the influence of working conditions and cultural factors on employees' dietary habits, which can guide more effective workplace health policies.

## Appendices

Questionnaire of lifestyle and dietary habits among automotive sector workers

1.    How old are you? _____ years old

2.    What is your marital status?

( ) Married ( ) Single ( ) Divorced

3.    What is your highest level of education completed?

( ) Primary school ( ) Middle school ( ) High school ( ) University

4.    What is your current occupation? _____

5.    How long have you been working in total? _____ years

6.    What are your average weekly working hours?

( ) 40 hours ( ) 45 hours ( ) More than 45 hours

7.    What is your height? _____ cm

8.    What is your weight? _____ kg

9.    Do you have any chronic diseases diagnosed by a healthcare provider?

( ) Yes __________ ( ) No

10.   Are you currently taking any medications on a regular basis?

( ) Yes ( ) No

11.   Do you have any hobbies that you engage in regularly (e.g., sports, crafts, and music)?

( ) Yes ( ) No

12.   Do you smoke cigarettes?

( ) Yes ( ) No

13.   Do you consume alcoholic beverages?

( ) Yes ( ) No

14.   Do you chew tobacco?

( ) Yes ( ) No

15.   Do you use vitamins or dietary supplements?

( ) Yes ( ) No

16.   Do you use artificial sweeteners (e.g., aspartame and sucralose)?

( ) Yes ( ) No

17.   How often do you consume red meat per week? ( ) None ( ) Less than one day per week ( ) 1-2 days per week ( ) 3-4 days per week ( ) 5-6 days per week

18.   How do you prefer your red meat to be cooked? ( ) Undercooked ( ) Medium cooked ( ) Well-cooked

19.   How often do you consume poultry meat per week? ( ) None ( ) Less than one day per week ( ) 1-2 days per week ( ) 3-4 days per week ( ) 5-6 days per week

20.   How often do you consume fish per week? ( ) None ( ) Less than one day per week ( ) 1-2 days per week ( ) 3-4 days per week ( ) 5-6 days per week

21.   How often do you consume vegetables per week? ( ) None ( ) Less than one day per week ( ) 1-2 days per week ( ) 3-4 days per week ( ) 5-6 days per week

22.   How much water do you drink daily? ( ) Less than one glass ( ) 1-2 glasses ( ) 3-4 glasses ( ) 5-7 glasses ( ) Eight or more glasses

23.   How much tea do you drink daily? ( ) Less than one glass ( ) 1-2 glasses ( ) 3-4 glasses ( ) 5-7 glasses ( ) Eight or more glasses

24.   How much coffee do you drink daily? ( ) Less than one cup ( ) One cup ( ) Two cups ( ) Three cups or more
